# Distant mood monitoring for depressive and bipolar disorders: a systematic review

**DOI:** 10.1186/s12888-020-02782-y

**Published:** 2020-07-22

**Authors:** A. S. J. van der Watt, W. Odendaal, K. Louw, S. Seedat

**Affiliations:** 1 Department of Psychiatry, Stellenbosch University, Tygerberg, South Africa; 2 Health Systems Research Unit, South African Medical Research Council, Cape Town, South Africa

**Keywords:** Affective disorder, Distant, Intervention, LMIC, Mood disorder, Monitoring, Task-shifting, South Africa

## Abstract

**Background:**

Broadening our knowledge of the longitudinal course of mood symptoms is cardinal to providing effective long-term treatments. Research indicates that patients with mental illness are willing to engage in the use of telemonitoring and mobile technology to assess and monitor their mood states. However, without the provision of distant support, adverse outcomes and events may be difficult to prevent and manage through self-monitoring. Understanding patient perspectives is important to achieving the best balance of self-monitoring, patient empowerment, and distant supporter involvement.

**Methods:**

This systematic review synthesises quantitative and qualitative evidence of the effectiveness and feasibility of daily/weekly/monthly remote mood monitoring that includes distant support in participants with mood disorders. Inclusion criteria comprised mood monitoring of mood disorder patients as main intervention, study design, method of monitoring, and presence of psychotherapy and psychoeducation. Effectiveness was defined by the change in depression and/or mania scores. Feasibility was determined on participant feedback and completion/attrition rates. Studies were assessed for quality using the Mixed Methods Appraisal Tool version 2018.

**Results:**

Nine studies of acceptable quality met the inclusion criteria. Distant mood monitoring was effective in improving depression scores but not mania scores. Feasibility, as measured through compliance and completion rates and participant feedback, varied.

**Conclusion:**

Distant mood monitoring with support may be a useful, acceptable, and feasible intervention for diverse groups of patients in terms of age and ethnicity. Further, it may be effective in improving symptoms of depression, increasing treatment adherence, and facilitating the prevention and management of adverse outcomes. As a task-shifting intervention, distant mood monitoring may help to alleviate the burden on mental health providers in developing countries.

## Background

Broadening our knowledge of the longitudinal course of mood symptoms is cardinal to understanding bipolar and unipolar depression, and other affective disorders (hereafter referred to as mood disorders) and providing long-term effective treatments. This includes patterns of chronicity, episodicity, relapse, and recurrence [1, 2], especially for bipolar disorder [3]. Such knowledge underlines the investigation of pathophysiological mechanisms, assists in guiding and optimising treatment (e.g. dose, duration [4]), and informs the development of novel and more effective treatments [5].

Research indicates that patients with psychiatric disorders readily engage in the use of telemonitoring [6] and mobile technology [7, 8] as forms of mood assessment, monitoring, and treatment; allowing for more regular data collection on mood trajectories. A systematic review of the validity of electronic self-monitoring of mood using information technology (IT) platforms in adults with bipolar disorder found evidence of their validity when compared to clinical rating scales for depression [9].

Both weekly telemonitoring and text messaging allow for improved access to professional care in patients with bipolar disorder [6, 10, 11] and may facilitate symptom improvement. For example, patients with bipolar disorder endorsed lower levels of illness experienced during facilitated integrated mood management [8]. Telemonitoring and text messaging to monitor patients’ mood fluctuations, though not cost-free, is far less expensive than traditional clinical interviews [8, 11]. Lastly, these interventions may assist in increasing treatment adherence which is of benefit as non-adherence is a major and costly concern in the treatment of mood disorders [11].

Whilst electronic self-monitoring and intervention programmes may promote patient self-management and empowerment, keeping some form of interaction with trained supporters (such as clinicians, counsellors, and researchers) is positively valued by patients and allows for a more personalised approach that improves efficiency [1, 12, 13]. Additionally, the prevention and management of adverse outcomes and events may be hampered without the proper involvement of clinicians [14] or other trained supporters. Further, involvement of trained supporters may indirectly increase the effectiveness of the intervention through the quasi-therapeutic experience [15]. Optimising treatment and minimising adverse events in distant mood monitoring programs requires an understanding of patients’ perspectives so that the best balance between self-monitoring, patient empowerment, and distant supporter involvement can be achieved [16]. Further, the COVID-19 pandemic has highlighted the need for a better understanding of telepsychiatry and distant interventions, which is corroborated by the April 2019 release of the World Health Organization (WHO) guideline on digital interventions for health system strengthening [17]. There is likely to be a surge of research in this area, as reflected in the 25 effectiveness reviews on digital health published by the Cochrane Library [18] and two overviews of reviews on digital health [19, 20] that jointly identified 29 systematic reviews, of which 17 were non-Cochrane reviews.

This systematic review evaluates the effectiveness and feasibility of distant mood monitoring, with support, in individuals with mood disorders.

### Objectives

We synthesised quantitative and qualitative evidence on the effectiveness and feasibility of daily/weekly/monthly remote mood monitoring in participants with any mood disorder (as defined above) by clinicians, lay counsellors, and researchers (hereafter referred to as distant supporters), or where regular feedback was provided by distant supporters in cases where mood states were self-assessed. Assessment of effectiveness was based on the change in depression and/or mania scores. Feasibility was determined according to completion/attrition rates and participant feedback. Studies were assessed for quality using the Mixed Methods Appraisal Tool (MMAT) version 2018 [21].

## Methods

This review is registered on PROSPERO (CRD42017057227).

### Literature search

The first and second authors searched the following databases to identify eligible articles:

i.) Academic search premier – EBSCOhost
ii.) PubMed – Medline
iii.) SAGE journals
iv.) Web of Science
v.) Cochrane

**Fig. 1:**
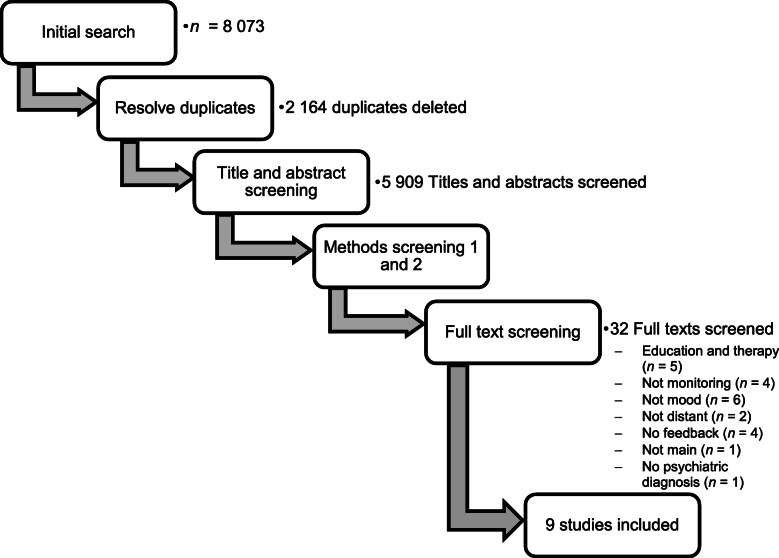
Screening process

Additionally, the reference lists of included studies were searched to identify potentially relevant studies that may have been missed by electronic searches [22]. After the first phase of the screening process (see Fig. 1), relevant articles to which we did not have full text access were flagged. These articles were requested through an inter-library loan process at Stellenbosch University.

### Search strategy

The following keywords (and MeSH terms) were used in searching for relevant literature:

- Telephonic OR telephone OR mobile phone OR cellular phone OR cell phone OR smartphone OR computer OR telecommunication OR electronic OR Skype OR pen-and-paper OR paper-and-pencil OR wearables OR mood charting
- AND
- Psychiatric Disorder OR mental disorder
- AND
- Monitoring OR remote monitoring OR distant monitoring OR no contact monitoring
- AND
- Clinician OR lay counsellor OR researcher OR therapist OR counsellor OR psychiatrist

### Eligibility criteria

Only peer reviewed studies published in English between 01 January 2000 and 26 September 2019, were considered for the review. Inclusion criteria were not limited to study setting or location.

#### Mood monitoring as the main intervention

For a study to be eligible, it had to primarily focus on the effectiveness of daily/weekly/monthly distant mood monitoring of participants. Monitoring occurring at intervals longer than once a month (e.g. every 3 months) were excluded. Studies where the mood monitoring was not deemed the primary focus (or intervention), were excluded.

Participants had to have a diagnosis of a mood disorder as defined by the Diagnostic and Statistical Manual of Mental Disorders [23, 24] or the International Statistical Classification of Diseases and Related Health Problems [25].

#### Study design

All quantitative studies were included as well as studies that qualitatively assessed participants’ perceived effectiveness, feasibility, and acceptability of distant mood monitoring offered by distant supporters. Case studies were also considered for inclusion. Systematic reviews and commentaries were excluded. Studies included in other systematic reviews that met our inclusion criteria were included, but not the systematic review itself.

#### Method of monitoring

Only studies in which the mood monitoring was done *distantly* were included, and the monitoring had to take place without any face-to-face contact (i.e. physical presence) between the study participant and the person conducting the distant mood monitoring. In the present review, *monitoring* refers to (i) supporters distantly monitoring participants’ mood (daily, weekly, bi-weekly, or monthly) and (ii) participants monitoring their own mood (daily, weekly, bi-weekly, or monthly). With reference to the former, the focus was on studies in which mood monitoring was done *distantly*, without any face-to-face contact (i.e. physical presence) between the patient and the person conducting the distant mood monitoring - studies where mood monitoring took place via telephone, internet, smartphone, and/or e-mail. This monitoring had to be done by a distant supporter. With reference to the latter, self- monitoring of participants’ own mood had to be accompanied by distant supporter feedback (daily, weekly, bi-weekly, or monthly). Studies that focused only on participants’ self-monitoring of mood states in the absence of distant supporter feedback were excluded as well as studies where feedback was only computer generated without the assistance of a distant supporter.

The decision to focus on mood monitoring conducted by a distant supporter (or which at least included some feedback by a distant supporter) was based on research indicating the effectiveness of participants being listened to [26], or simply talking to a researcher interested in what they have to say [27]. As such, mood monitoring that involves contact, albeit distant, with a distant supporter may have therapeutic benefits in and of itself [28]. There is also some evidence that participants often take part in research studies, such as mood monitoring, to access the aforementioned benefits [29].

#### Psychotherapy and psychoeducation

Studies in which the monitoring co-occurred with psychotherapy or psychoeducation as part of the intervention, for example where telephonic mood monitoring was followed by telephonic psychotherapy, [30] were excluded. This was done to differentiate between the effectiveness of *mood monitoring* of itself versus psychotherapy or psychoeducation delivered distantly.

### Screening process

Articles identified through the search (*N* = 8073) were exported to Rayyan [31] where the first and second authors independently assessed their eligibility using the blind function. During each screening phase (see Fig. 1), the authors indicated the main reason for exclusion using the “Reason” function in Rayyan. During each subsequent phase, the main exclusion reason applied in the previous phases could still apply (e.g. if a duplicate was missed during Phase 1, it could still be indicated as the main reason for exclusion during Phase 2 – Phase 5). The phases, with the main exclusion reasons of each, are described next.

First, duplicates (*n* = 2164) were removed. Second, titles and abstracts were screened. Main exclusion reasons included: (i) Review articles (*n* = 725; including systematic reviews, literature reviews, narrative reviews, or commentary articles); (ii) Posters and presentations (*n* = 699; including poster/oral presentations at conferences and collections of abstracts); (iii) Animal studies (*n* = 44); (iv) Protocols (*n* = 274; including published protocols and trial registrations not including results); and (v) No psychiatric diagnosis (*n* = 3764; including articles where participants did not have a clear psychiatric diagnosis).

Since limited details are provided in an abstract, a third phase was added in which the methods, but not the full texts, were superficially screened. Exclusion reasons for Phase 3 included: (i) Monitoring frequency (*n* = 1674; including articles where monitoring was not done daily, weekly, bi-weekly, or monthly); (ii) Not studies on mood (*n* = 556; including, for example, articles where alcohol intake or medication adherence was monitored, but not mood symptoms, or where mood was monitored as part of the larger study but findings did not include mood data); and (iii) Not studies on distant monitoring (*n* = 126; including articles where the intervention included face-to-face visits with clinicians (excluding treatment as usual) or research staff).

The fourth phase included a more thorough screening of the methodology section. During this phase, articles were excluded for the following reasons: (i) No feedback provided (*n* = 75; including articles where there was no feedback/support as per the criteria above); (ii) Education and therapy (*n* = 56; where psychoeducation or psychotherapy formed part of the intervention); and (iii) Not main intervention (*n* = 6; where monitoring was not the main intervention; for example where a new drug was being tested). These exclusion reasons were only added at a later phase since a much more thorough reading of the methodology section was needed.

Lastly, the full text of the remaining articles (*n* = 32) were screened to determine eligibility. Of the full text articles screened only 9 were included. Reasons for exclusion included: (i) Education and therapy (*n* = 5); (ii) Not monitoring (*n* = 4); (iii) Not mood (*n* = 6); (iv) Not distant (*n* = 2); (v) No feedback (*n* = 4); (vi) Not main (*n* = 1); and (vii) No psychiatric diagnosis (*n* = 1).

### Quality assessment

The first and second authors independently assessed the quality of the included studies using the Mixed Methods Appraisal Tool (MMAT) version 2018 [21, 32]. However, since two of the included studies [5, 28] were authored by the first author, a third independent researcher was asked to assess the quality of these two studies.

### Data collection

The first author extracted the relevant information from the included studies, and the second author corroborated the information. The extracted data included study design, setting, sample, mood disorder, method of monitoring, any additional information deemed important, and study findings.

### Outcomes

The two main outcomes for which the data were sought were (i) effectiveness, and (ii) feasibility of daily/weekly/monthly remote/distant mood monitoring by distant supporters of participants with mood disorders.

## Results

### Eligible papers

Nine articles [5, 28, 33–39] met the inclusion criteria for this systematic review. Mood monitoring studies were conducted in Chile [35], Denmark [33, 34], South Africa [5, 28], and the United States of America [36–39]. Details regarding study designs and monitoring procedures are presented in Table 1. Most studies used quantitative methods [5, 35–40], with only two studies [28, 34] including a qualitative component (semi-structured interviews). Details regarding outcome measures, effectiveness, adverse events, and completion rates are presented in Table 2. Information regarding Population, Intervention, Comparison, Outcome, and Time (PICOT) is embedded in both Table 1 (P, I, C, T) and Table 2 (C, O).

**Table 1:** Description of study design and monitoring procedures

Authors	Study Design	Sample Characteristics (Population and Comparison)			Mood Monitoring Information (Intervention and Time)				
		Mood Disorder	Sample Size	Mean Age (Range; SD)	Monitoring	Feedback	Frequency	Total Length (weeks)	Start of monitoring
**Faurholt-Jepsen, Vinberg et al., 2015**^a^	RCT single blind	BD	Intervention: *n* = 3 Placebo-control group provided with mobile phone for daily use: *n* = 39	Intervention: 29.1 (NS; 7.5 years) Control: 29.5 (NS; 9.4 years)	Smart phone application (MONARCA)	Contacted by nurse when necessary; graphic visualization	Daily	24	NS: Outpatient population
**Lauritsen et al., 2017** [36]	Single arm observational	MDD	*N* = 45	35.9 (NS; 10.8 years)	Online (Daybuilder webpage)	Telephonic, weekly; graphic visualization	Twice, daily	4	NS
**Martinez et al., 2018**	Assessor-blind cluster RCT	MDD	Intervention: *n* = 65 EUC control: *n* = 78	Intervention: 15.2 (NS; 1.5 years EUC control: 15.6 (NS; 1.7 years)	Telephonic	Telephonic	At weeks 1, 2, 3, 6, 9	12	NS
**Piette et al., 2013** [37]	Observational	Depressive Disorder	*N* = 387	NS^b^ (21–66+ years; NS)	IVR calling system	Clinical teams with actionable feedback and informal caregivers with feedback.	Week 1–6: Weekly with option to reduce to monthly if depression scores were mild enough. Can revert back to weekly at any time.	21–48 (median = 25)	NS
**Ross et al., 2008** [38]	RCT	Minor Depression	Intervention: *n* = 130 TAU control: *n* = 93	Intervention: 59.8 (NS; 14.6 years) Control: 58.5 (NS; 17.7 years)	Telephonic	Telephonic	Weekly	8	NS: Outpatient population
**Van der Watt, Roos et al., 2018**	Mixed-method	MDD and BD	Interviews: *n* = 37	Interviews: 35.76 (18–53; 10.8 years)	Telephonic	Telephonic	Weekly	26	1 week post-discharge
**Van der Watt, Suryapranata et al., 2018**	Longitudinal	MDD and BD	*N* = 61	35.3 (18–53; 10.2 years)	Telephonic	Telephonic	Weekly	26	1 week post-discharge
**Yeung et al., 2012**^a^ [39]	Non-randomised controlled trial	MDD	Intervention: *n* = 503 Control: *n* = 412	Intervention: 46.6 (18–65+; 15.0 years) Control: 45.3 (18–65+; 15.4 years)	Telephonic (COMET)	Telephonic	Intervention: Monthly Control: At 3 and 6 months	24	Following diagnosis by physician
**Zulueta et al., 2018**^**b**^ [34]	Observational	BD	*N* = 16	48.67 (NS; 9.63 years).	Telephonic (BiAffect)	Telephonic	Weekly	8	NS

*AD* Anxiety Disorder, *BD* Bipolar Disorder, *COMET* Clinical Outcomes in Measurement-based Treatment, *EUC* Enhanced Usual Care, *IVR* Interactive Voice Response, *MDD* Major Depressive Disorder, *NS* Not specified, *RCT* Randomized Controlled Trial, *TAU* Treatment As Usual

^a^Demographic data only presented for participants who completed the study

^b^There were a broad range of ages, with 31% being 21 to 45 years old and 12% being 66 years or older

**Table 2:** Description of outcome measures, effectiveness, adverse events, and completion rates

Authors	Primary Outcome Measures (Comparison and Outcome)				Feasibility and Effectiveness (Outcome)	
	Depression scale/instrument	Mania	Frequency	Adverse events reported	Completion Rate	Effective
**Faurholt-Jepsen, Vinberg et al., 2015**^**c**^	HAMD-17	YMRS	Monthly for 6 months	Trained nurse contacted participant if deterioration in symptoms detected. Results NS	Intervention: 33/39 = 82.62% Control: 34/39 = 87.18%	No significant improvement in HAMD-17 or YMRS scores.
**Lauritsen et al., 2017** [36]	HAMD-17; MINI; MDI	NA	Baseline; at 4 weeks	5 participants were readmitted to an inpatient ward due to worsening depression (self-monitoring continued)	General: 34/45 = 76%	59% of participants believed that the system could detect a relapse, 50% believed that the system could influence the course of their illness, and 50% felt that the system had covered their needs for self-monitoring. No significant improvement in self-assessed mood scores. Significant improvement in HAMD-17 and MDI scores.
**Martinez et al., 2018**	BDI	NA	Baseline; at 12 weeks	NS	Intervention: 65/65 = 100% EUC control: 73/78 = 83.49%	Participants rated the intervention as 6/7 (88.57%) in terms of both usefulness and comfort. Clinicians rated the intervention for usefulness for clinical work (90%), usefulness for patients (92.86%), and comfort (85.71%). No significant differences were observed across arms at 12-week follow-up in terms of depressive symptomology. However, regression analysis indicated (i) for each extra point in baseline BDI scores, a reduction of 0.5 points in BDI scores at 12 weeks; and (ii) for each additional point in satisfaction with the psychological care received, a reduction of 4.3 points in BDI scores at 12 weeks.
**Piette et al., 2013** [37]	PHQ-9	NA	Week 1–6: Weekly with option to reduce to monthly if depression scores were mild enough. Could revert to weekly at any time.	Alerts generated for suicidal ideation, poor medication adherence, and increased depressive symptom severity. Alerts were triggered at a rate of 4.9 per 100 person-weeks of participation.	11% attrition in first 6 months; 68% assessment completion	NS
**Ross et al., 2008** [38]	PHQ; MINI	NA	Baseline; at 6 months	Participants (37.7%) referred to the behavioural health specialist.	Intervention: 96/130 = 73.85% Control: 72/93 = 77.42%	Intervention group had less (not significantly) depression symptoms and diagnoses at 6-months follow-up than control group.
**Van der Watt, Roos et al., 2018**	QIDS	ASRM	Weekly for 26 weeks	Participants reported negative (10.8%) and apprehensive (16.2%) experience of baseline assessment.	Interviews conducted regarding effectiveness: 60.7%	Majority of participants interviewed (86.5%) reported that they found the mood monitoring helpful.
**Van der Watt, Suryapranata et al., 2018**	QIDS	ASRM	Weekly for 26 weeks	NS	45.9%	Significant improvement in QIDS scores. No significant difference in ASRM scores.
**Yeung et al., 2012**^a^‑ [39]	PHQ-9; PGI-S	NA	Monthly	Physicians were sent reports on participants’ PHQ scores. 273 PHQ-9 responses endorsing thoughts of self-harm were reported to physicians.	Intervention: 364/503 = 72.37% Control: 278/412 = 67.48%	45% achieved remission by the end of the study, with the intervention group being significantly more likely to achieve remission. 53.9% fulfilled the response criterion (50% + reduction in PHQ-9 scores), with the intervention group being significantly more likely to achieve response.
**Zulueta et al., 2018** [34]	HAMD-17	YMRS	Weekly	NS	Participation varied in terms of the number of weeks that had any keyboard activity, with an average of 4.69 (3.05) weeks. Only 9 participants (9/16 = 56.25%) complete at least 4 weeks.	Decrease in HAMD-17 scores: Week 1 = 11.90 (3.17); Week 8 = 11.11 (5.49). Significance not reported. Decrease in YMRS scores: Week 1 = 7.56 (5.00); Week 8 = 6.67 (4.03). Significance not indicated.

*ASRM* Altman Self-Rating Mania Scale, *BDI* Beck Depression Inventory, *HAMD-17* Hamilton Depression rating scale, *MDI* Major Depression Inventory, *MINI* Mini-International Neuropsychiatric Interview, *NA* Not Applicable, *NS* Not Specified, *PGI-S* Patient Global Impression Severity, *PHQ* Personal Health Questionnaire, *QIDS* Quick Inventory of Depressive Symptomatology, *YMRS* Young Mania Rating Scale

^a^Demographic data only presented for participants who completed the study

^b^Demographic data only presented for participants who completed the study

^c^Demographic data only presented for participants who completed the study

### Quality of the included papers

Table 3 presents a summary of the MMAP quality assessment of the included articles. In general, the quality was deemed acceptable based on the different study designs. Methodological concerns related to sample representativeness [36], complete outcome data [38], control of confounding variables [39], a rationale for the use of a mixed method study design [28], and outcome assessors being blinded to the randomized controlled trial (RCT) intervention [37].

**Table 3:** MMAP quality appraisal of included articles

**MMAP Quantitative non-RCT quality appraisal**					
	**Lauritsen et al., 2017** [36]	**Piette et al., 2013** [37]	**Van der Watt, Suryapranate et al., 2018**	**Yeung et al., 2012** [39]	**Zulueta et al., 2018** [34]
1. **Are the participants representative of the target population?**	Yes: Patients suffering from MDD were recruited to participate post discharge. The authors acknowledge that patients referred to the facility may belong to a more severely depressed subset of inpatients.	No: The included participants were not representative of the target population in terms of race, sex, and education.	Yes: Inpatients with a primary mood or anxiety disorder were recruited pre-discharge.	Partially: Participants were recruited based on a physician’s diagnosis of MDD. The authors acknowledge the influence physician selection bias and that standard diagnostic criteria of patients may not have been met.	Partially: Patients suffering from bipolar disorder were recruited to participate in the study. The authors acknowledge that the sample is not representative of the target population in terms of sex.
2. **Are the measurements appropriate regarding both the outcome and intervention (or exposure)?**	Yes: The intervention involved monitoring mood and quality of sleep, daily, using a Visual Analog Scale (VAS). Depression outcome was measured using the well-established HAM-D-17 measure.	Yes: The intervention involved monitoring mood and medication adherence using Interactive Voice Response (IVR) technology. Depression outcome was measured using the well-established PHQ-9.	Yes: The intervention involved interepisodal telephonic mood monitoring and the outcomes were measured weekly for 26 weeks using established tools, the ASRM and QIDS.	Yes: The intervention involved monthly telephonic monitoring of depression symptom severity using the well-established PHQ-9.	Partially: The intervention involved weekly telephonic mood monitoring using well-established measures (HAM-D-17; YMRS) and ecological monitoring using keystroke data. The use of keystroke data as indicators of mood symptoms is partially motivated in the introduction section; yet well-established evidence is lacking.
3. **Are there complete outcome data?**	Yes: Mood, sleep, and activity outcomes were analysed using available data from all included patients. The completion rate is relatively high (76%) and the authors clearly indicate the reasons for attrition. Outcome data is reported for all measurements used.	Yes: Mood and medication adherence were analysed using available data from all included participants. Reasons for attrition were not provided.	Yes: Although the drop-out rate was quite high, results showed a significant decline in depression scores. ASRM scores were not indicative of significant mania and the authors also reported data for suicidality.	No: In both the intervention and control group, data were excluded from analysis due to the lack of an interview at 6 months or a too low PHQ-9 score at baseline.	Partially: Missing data were handled with pairwise deletion.
4. **Are the confounders accounted for in the design and analysis?**	Partially: Analyses model of mood included time, sleep-onset, sleep-offset, sleep quality, activity, and interactions between sleep-onset and day, sleep-offset and day, sleep quality and day, and activity and day. The authors acknowledged confounders that may have had an influence on the data, was not included in the present study data.	Partially: Analyses of completion rates controlled for demographic characteristics, measures of baseline vulnerability, baseline depression scores, and weeks of follow-up. No other confounders are mentioned.	Partially: The authors collected data on traumatic childhood experiences but did not state if these were accounted for as confounders in the data analysis. No other confounders are mentioned.	Partially: Covariates to control for patient demographics and clinical history were included in the logistic regression models. The authors acknowledge that may have had an influence on the data, was not included in the present study data.	No: Possible confounders were not clearly identified, or how they were controlled for.
5. **During the study period, is the intervention administered (or exposure occurred) as intended?**	Yes: The intervention was administered as intended.	Yes: The intervention was administered as intended.	Yes: The intervention was administered as intended.	Yes: The intervention was administered as intended	Yes: The intervention was administered as intended
**MMAP Quantitative RCT quality appraisal**				**MMAP Mixed Methods quality appraisal**	
	**Faurholt-Jepsen et al., 2015**	**Martinez et al., 2018**	**Ross et al., 2018** [38]		**Van der Watt, Roos, et al., 2018**
1. **Is randomization appropriately performed?**	Yes: Participants were randomized with a balanced ration of 1:1 to receive either an intervention Android smartphone (the intervention group) or a control Android smartphone (the control group) for a 6-month trial period.	Yes: Randomization was conducted using computer-generated random numbers	Unclear: Consented clinicians were randomly assigned to either usual care or close monitoring. Randomization was stratified by clinic. However, it is unclear how participants were randomized.	1. **Is there an adequate rationale for using a mixed methods design to address the research question?**	No: There is no rationale provided
2. **Are the groups comparable at baseline?**	Yes: Randomization was stratified on age (< 29 or ≥ 29 years) and former hospitalization (yes/no) since these were considered to be possible prognostic variables, and a fixed block size of 10 within each stratum was used.	Yes: Participants’ baseline sociodemographic characteristics were similar, with the exception of socioeconomic status (*p* = 0.03)	Partial: See Table 1 in the article. At baseline the two groups statistically differed in terms of sex, finance, and the experience of a disturbing traumatic event.	2. **Are the different components of the study effectively integrated to answer the research question?**	Yes: The qualitative and quantitative components complement each other and function well as a unified whole to answer the research question.
3. **Are there complete outcome data?**	Yes: 82.62% of the intervention group data, and 87.18% of the control group data could be analysed.	Yes: It appears as if all the data mentioned in the measurement section is reported	Yes: 72.31% of the intervention group data, and 77.42% of the control group data could be analysed.	3. **Are the outputs of the integration of qualitative and quantitative components adequately interpreted?**	Yes: The qualitative component provides detailed evidence for acceptability and perceived effectiveness of mood monitoring and reasons for participant drop-out. This information is effectively supported by quantitative data including baseline assessment and post-discharge assessment using established questionnaires.
4. **Are outcome assessors blinded to the intervention provided?**	Partially: Due to the type of intervention, this trial was single-blinded since blinding of the participants, the clinicians, and the study nurse handling the intervention was not possible.	Yes: Patient baseline data and outcomes at 12-week follow-up were evaluated via telephone by a trained consultant who was blinded to treatment allocation.	No: Due to the nature of the intervention, blinding was not possible.	4. **Are divergences and inconsistencies between quantitative and qualitative results adequately addressed?**	Unclear: There appears to be no mention of any divergence or inconsistencies between quantitative and qualitative results.
5. **Did the participants adhere to the assigned intervention?**	Yes: A total of 3.7% of participant visits were missing (3.6% in the intervention group and 3.8% in the control group) due to participants not attending.	Partially: No participants assigned to the intervention were lost to follow-up. However, only one-third of the patients displayed an adequate adherence to the pharmacological treatment	Unclear: Follow-up data is reported for 75.3% (6 months) for the participants in general. However, it is not clear how many completed the weekly assessments.	5. **Do the different components of the study adhere to the quality criteria of each tradition of the methods involved?**	Yes: It is reflected in the analysis and reporting of the data.

*ASRM* Altman Self-Rating Mania scale, *HAM-D-17* Hamilton Depression rating scale, *MDD* Major Depressive Disorder, *PHQ* Patient Health Questionnaire

### Descriptive data: demographic information, mood disorder, and assessments

As indicated in Table 1, the majority of participants in each study were female, ranging from 55.6% [34] to 89.2% [28], with the exception of the study by Ross and colleagues (2008) where females were the minority (6.7%). Age of participants varied widely from as young as a mean age of 15.2 years [35] to above 66 years old [36]. Three studies [34, 39, 40] did not provide details on ethnicity. Two of the studies conducted in the USA included mainly white participants, ranging between 75.4% [38] and 90% [36]. Similarly, a Chilean study included mainly white (83.2%) participants [36]. The remaining studies [5, 28, 35, 38] included diverse ethnic groups including African American, black, coloured (mixed race), Hispanic, and Mapuche participants.

Across the studies, included participants predominantly had depressive disorders [34–38]. Two studies included both depressive and bipolar disorders [5, 28], and two included only bipolar disorder participants [39, 40].

Outcome measures for depression included the Beck Depression Inventory (BDI) [35], the Hamilton Depression rating scale (HAM-D) [34, 39, 40], the Major Depression Inventory (MDI) [34], the Mini-International Neuropsychiatric Interview (MINI) [34, 37], the Patient Health Questionnaire (PHQ) [36–38], the Patient Global Impression Severity scale (PGI-S) [38], and the Quick Inventory of Depressive Symptomatology (QIDS) [5, 28]. Outcome measures for mania included the Altman Self-Rating Mania scale (ASRM) [5, 28] and the Young Mania Rating Scale (YMRS) [39, 40]. Only three studies specified the time at which distant mood monitoring had commenced in relation to the course of the disorder. For these studies, distant mood monitoring was initiated at 1 week post-discharge [5, 28] or following diagnosis [38]. The remaining studies indicated that outpatients were recruited [34–37, 39, 40].

### Feasibility

For this systematic review, feasibility was based on compliance and completion rates, and on feedback provided by participants. For RCTs included in this review, control group completion rates ranged between 67.48% [38] and 87.18% [40]; while intervention group completion rates ranged between 72.37% [38] and 100% [35]. For the other included studies, completion rates ranged between 45.9% [5] and 76% [34]. In the present review, only one study [28] reported on participants’ subjective experiences of the acceptability of distant mood monitoring with support, and found high acceptability. However, Van der Watt, Roos, and colleagues (2018) also noted that some participants reported negative (10.8%) and apprehensive (16.2%) experiences of the baseline assessment prior to the commencement of mood monitoring. The negative experiences of baseline assessments, which included two trauma questionnaires, included feeling “… nervous … like a rat in a trial” speaking to strangers (researchers) about personal experiences and feelings, and “… experience [ing] the reality again” of past experiences [28]. None of the included studies reported on adverse events directly linked to the mood monitoring.

### Effectiveness

We defined effectiveness (or a lack thereof) in terms of an increase and/or decrease in depression and/or mania scores on a rating scale, as well as participants’ self-reports of the helpfulness of the mood monitoring.

One study [36] did not specify whether there was an increase or decrease in depression scores. Two studies [37, 40] reported no significant decrease in depression scores, while three studies [5, 35, 38] reported a significant decrease in depression scores following distant mood monitoring. Qualitatively, participants reported that distant mood monitoring was helpful [28]. However, it should be noted that this positive feedback was not limited to participants with major depressive disorder. Nonetheless, distant mood monitoring with support appears to be effective in decreasing depression symptoms.

Distant mood monitoring does not seem to be effective in significantly decreasing mania scores [5, 40] even though the majority of participants (86.5%) reported that the distant mood monitoring was helpful [28]. This was, however, not limited to participants with bipolar disorder. Further, Zulueta and colleagues (2018) reported a decrease in mania scores, yet they did not specify whether this decrease was statistically significant or not.

Throughout mood monitoring, adverse events may occur that are not necessarily because of the mood monitoring. In terms of such adverse events, four studies [5, 28, 35, 39] did not report any adverse events or procedures to manage them. One study [40] indicated that trained nurses would contact participants should they detect a deterioration in symptoms, however, the authors did not indicate how often this occurred. Four studies specified adverse events that occurred during mood monitoring and/or procedures to manage them: readmission [34]; alerts generated for suicidal ideation, poor medication adherence, and increased depressive symptom severity [36]; referral to the behavioural health specialist following persistent or worsening depressive symptoms [37]; and thoughts of self-harm reported to physicians [38].

## Discussion

Most of the studies included in the review were conducted in high income countries (*n* = 6). This highlights the need for more research to be conducted in developing countries. Due to the high prevalence of depressive and bipolar disorders in low- and middle-income countries (LMIC) (see for example [41–43]), combined with the lack of mental health providers (see for example [44–46]), task-shifting has become increasingly important (see for example [47, 48]). Distant mood monitoring interventions with adjunctive support (which could be provided by trained lay counsellors and researchers) may help to alleviate the burden on mental health providers in LMIC.

Apart from the study by Ross and colleagues (2008), participants were mostly female. This likely reflects the higher prevalence rate of depressive and bipolar disorders in women [49, 50]. The wide age range and ethnic diversity of participants suggests that distant mood monitoring, with feedback, has wide applicability.

Included studies mainly focused on depressive disorders (*n* = 5). This limits the generalisability of conclusions that can be made to patients with bipolar disorder. Further research is needed in this regard. Studies used a variety of instruments to determine depression (BDI, HAM-D, MINI, PHQ, PGI-S, and QIDS) and mania outcomes (ASRM, YMRS), which suggests that a number of measures may have applicability in distant mood monitoring. It should be noted that differences in outcome measures (i.e., use of different rating scales) across the studies may have impacted on responses [51] and outcomes [52, 53]. Only three studies indicated that distant mood monitoring commenced 1 week post-discharge (*n* = 2) or upon diagnosis (*n* = 1). To arrive at more accurate conclusions about the effectiveness of mood monitoring with support, it is important that future publications include details on the baseline timepoint in relation to the illness course. A better understanding of the course of depressive and bipolar disorders, especially following discharge from hospital, may facilitate early intervention and the scheduling of appointments, particularly in low-resourced settings.

### Feasibility

In general, distant mood monitoring with support was deemed feasible, with completion rates that are similar to those of distant self-mood monitoring interventions that did not include support [9]. Specifically, in their systematic review of distant self-mood monitoring, Faurholt-Jepsen and colleagues (2016) reported completion rates of between 42.1% [54] and 93.9% [55, 56]. Further, distant mood monitoring with support was highly acceptable to participants, while some participants reported negative or apprehensive experiences at the baseline assessment. However, reporting bias should be considered as qualitative subjective reports were largely obtained from participants who completed the study, with only a few participants who dropped out of monitoring reporting their experiences [28]. None of the other included studies reported on adverse events or side effects of the mood monitoring itself. Further research is needed to determine any negative effects that mood monitoring may have.

### Effectiveness

Distant mood monitoring with support was not effective in decreasing symptoms of mania. However, it does appear to be effective in decreasing depression symptoms. Thus, distant mood monitoring – as a task-shifting, relatively low-cost intervention – may be especially relevant in LMIC to assist in the treatment of depression. Considering the lack of information in this regard, the optimal time point at which to commence distant mood monitoring has yet to be determined.

Further, distant mood monitoring with support appeared to be effective in the timeous reporting of adverse events, such as symptom deterioration [34, 36, 40], suicidal ideation, poor medication adherence [36], and thoughts of self-harm [38]. This allowed for prompt intervention including readmission [34] and referral to behavioural health specialists [37]. These findings support previous research that distant mood monitoring may assist in improving treatment adherence [11]. Specifically, the involvement of distant supporters may facilitate the prevention and management of adverse outcomes [14]. Since treatment non-adherence is a major obstacle in the effective treatment of depressive and bipolar disorders [57, 58] the value of interventions that improve treatment adherence, such as distant mood monitoring, cannot be overstated. Furthermore, research has indicated that the level of motivation and engagement by a therapist [59] or supporter is correlated with treatment adherence. This strengthens the case for distant mood monitoring with support rather than mood monitoring without support.

### Limitations and recommendations

The present findings are based on a small (*n* = 9) number of published articles. Further, only four studies included control groups which play an important role in results interpretation. Studies with control groups, are recommended. The heterogeneity of study design and loose definitions of feasibility and effectiveness are additional limitations. Changes in depression and mania scores may not necessarily correlate with improvement in quality of life or functioning. Also, the databases that we searched did not include PsychInfo as our university does not have a license for this database. Strengths of this systematic review include two independent systematic searches as well as the use of a second, blinded, author screening process.

Distant mood monitoring with distant supporter feedback is an appealing intervention, particularly in low resource settings and during times when face-to-face contact is restricted, such as the COVID-19 crisis. Further research is need to better understand the role of distant mood monitoring with distant support and to confirm their feasibility and effectiveness in routine clinical care across different settings. This includes a better understanding of the timing of the intervention in terms of phase of illness, the potential harmful effects of regular mood monitoring with support, and barriers to the use and implementation of such monitoring systems.

More rigorous mood monitoring studies are needed to draw more definitive conclusions. These studies should provide more detail on (i) mechanisms of monitoring (for example what the feedback entails), (ii) mood trajectories (e.g. at different time-points instead of only at baseline and endpoint), (iii) adverse events related/unrelated to the mood monitoring itself, (iv) the experiences and perceptions of the participants during mood monitoring, and (v) the quality of life impact of treatment.

## Conclusion

This systematic review focused on the effectiveness and feasibility of daily/weekly/monthly remote mood monitoring in participants with any mood disorder by distant supporters or where regular feedback was provided by distant supporters in cases where mood states were self-assessed. Nine studies were found to be eligible for inclusion. Given the differences in sample characteristics, methodology, and outcome measures it is difficult to draw comparisons and definitive conclusions across the studies. However, we tentatively conclude that distant mood monitoring with support may be a useful and acceptable intervention for a diverse population in terms of age and ethnicity. Feasibility, as measured through completion rates and subjective feedback, is deemed acceptable. Further, distant mood monitoring with support may be effective in improving depression symptoms, increase treatment adherence, and facilitate the prevention and management of adverse outcomes. As a task-shifting intervention, distant mood monitoring may help to alleviate the burden on mental health providers in developing countries. These interventions also have appeal in a time when face-to-face contact is restricted and there is an expected increase in burden on the mental health system.

## Data Availability

Only summarized data from published articles are presented in this manuscript. The published articles are available from the respective authors and journals.

## Abbreviations

AD: Anxiety disorder
BD: Bipolar disorder
ASRM: Altman self-rating mania scale
BDI: Beck depression inventory
COMET: Clinical outcomes in measurement-based treatment
EUC: Enhanced usual care
HAMD-17: Hamilton depression rating scale
IT: Information technology
IVR: Interactive voice response
MDD: Major depressive disorder
MDI: Major depression inventory
MINI: Mini-International Neuropsychiatric Interview
NA: Not applicable
NS: Not specified
PGI-S: Patient global impression severity
PHQ-9: Personal health questionnaire
QIDS: Quick inventory of depressive symptomatology
RCT: Randomized controlled trial
TAU: Treatment as usual
YMRS: Young Mania Rating Scale
